# Dysbiosis of the Gut Microbiome in Lung Cancer

**DOI:** 10.3389/fcimb.2019.00112

**Published:** 2019-04-18

**Authors:** He Zhuang, Liang Cheng, Yao Wang, Yu-Kun Zhang, Man-Fei Zhao, Gong-Da Liang, Meng-Chun Zhang, Yong-Guo Li, Jing-Bo Zhao, Yi-Na Gao, Yu-Jie Zhou, Shu-Lin Liu

**Affiliations:** ^1^Systemomics Center, College of Pharmacy, and Genomics Research Center (State-Province Key Laboratories of Biomedicine-Pharmaceutics of China), Harbin Medical University, Harbin, China; ^2^HMU-UCCSM Centre for Infection and Genomics, Harbin Medical University, Harbin, China; ^3^Translational Medicine Research and Cooperation Center of Northern China, Heilongjiang Academy of Medical Sciences, Harbin, China; ^4^College of Bioinformatics Science and Technology, Harbin Medical University, Harbin, China; ^5^Department of Colorectal Cancer, The 2nd Affiliated Hospital of Harbin Medical University, Harbin, China; ^6^Department of Epidemiology, Public Health School, Harbin Medical University, Harbin, China; ^7^Department of Infectious Diseases, The First Affiliated Hospital, Harbin Medical University, Harbin, China; ^8^Department of Respiration, The Third Affiliated Hospital of Harbin Medical University, Harbin, China; ^9^Department of Microbiology, Immunology and Infectious Diseases, University of Calgary, Calgary, AB, Canada

**Keywords:** lung cancer, gut microbiota, next generation sequencing, 16S rRNA, microbial diversity, biomarkers

## Abstract

Lung cancer (LC) is one of the most serious malignant tumors, which has the fastest growing morbidity and mortality worldwide. A role of the lung microbiota in LC pathogenesis has been analyzed, but a comparable role of the gut microbiota has not yet been investigated. In this study, the gut microbiota of 30 LC patients and 30 healthy controls were examined via next-generation sequencing of 16S rRNA and analyzed for diversity and biomarkers. We found that there was no decrease in significant microbial diversity (alpha diversity) in LC patients compared to controls (*P* observed = 0.1422), while the composition (beta diversity) differed significantly between patients and controls (phylum [stress = 0.153], class [stress = 0.16], order [stress = 0.146], family [stress = 0.153]). Controls had a higher abundance of the bacterial phylum *Actinobacteria* and genus *Bifidobacterium*, while patients with LC showed elevated levels of *Enterococcus*. These bacteria were found as possible biomarkers for LC. A decline of normal function of the gut microbiome in LC patients was also observed. These results provide the basic guidance for a systematic, multilayered assessment of the role of the gut microbiome in LC, which has a promising potential for early prevention and targeted intervention.

## Introduction

Lung cancer (LC) is one of the deadliest malignancies, which has growing morbidity and mortality worldwide. It poses an enormous threat to the human health (Torre et al., 2015). Research on LC genetics and biology has opened opportunities for novel therapeutic strategies against the disease (Allison, 2015; Chowdhury et al., 2018; Hendriks and Besse, 2018; Herbst et al., 2018; Lissanu Deribe et al., 2018; Wei et al., 2018), but the knowledge about the etiology remains incomplete, making precision treatment or prevention a moving target. To date, the major etiological causes or risk factors facilitating the pathogenesis of cancers have been mostly focused on genetic susceptibility and carcinogenic environments (Addario, 2014; Gibbons et al., 2014; Liu et al., 2014), but people with high genetic or environmental risk factors may not develop the malignancies even at advanced ages. Conversely, in many cancer patients, clear familial or environmental risk factors are often non-traceable. These facts indicate the existence of additional major factors that influence the onset and development of cancers.

Over the past few decades, numerous discoveries have been reported regarding the gut microbiome for its roles in diseases with a particular focus on cancers (Jobin, 2012; Schwabe and Jobin, 2013; Gagliani et al., 2014). The diverse microbes in the human gut, 10-fold more than the total cells of the human host, with millions of total non-redundant genes, maintain a dynamic stable and healthy microenvironment inside the host. In cancer patients, the composition of the gut microbiota often becomes radically different from that in healthy individuals, for example, increased *Porphyromonas, Enterococcus, Streptococcus*, or *Peptostreptococcus* and decreased *Roseburia* or other beneficial microbes, such as butyrate-producing bacteria of the family *Lachnospiraceae* in cases of colorectal cancer (CRC) (Wang et al., 2012; Nakatsu et al., 2015). Meanwhile, the relative ratios and abundance of the resident microbes may also be directly associated with cancer, which have also been reported in CRC patients, such as *Streptococcus bovis* biotype I (Boleij et al., 2011), *E. coli* harboring *pks* (Cuevas-Ramos et al., 2010), and *Bacteroides fragilis* secreting DNA-damaging toxins (Toprak et al., 2006; Wu et al., 2009). Bacteria that are protective against cancer are also documented, such as *Lactobacillus rhamnosus* GG and *Lactobacillus acidophilus* (Neish, 2009; Verma and Shukla, 2013). In a recent study, we identified and characterized a large number of highly diverse anticancer bacteria from the gut of healthy individual, which had potent suppressive effects against a broad spectrum of cancer cell types *in vitro* and stopped the growth of tumors in a mouse model of human ovarian cancer. In this cancer model, the metastasized cancer cells were also cleared by intratumoral administration of the bacterial culture supernatant (Zhou et al., 2017). The highly effective anticancer activities of such commensal microbes have been detected in most participants of different age groups, demonstrating the existence of a strong cancer-defensive system parallel to the immune system in the human body (Zhou et al., 2017). Additionally, the high diversity of the cancer-suppressing bacteria targeting different cancer types suggests personalized anticancer microbial allies within individual hosts, which may confer different levels of resistance against or susceptibility to specific cancer types. Such speculations point to a possibility that cancers in a particular organ may have certain common features in the microbiome of the patients.

However, for LC, previous analyses focus on the relationship between LC and lung microbiome, where the microbes have direct contact with the lung tissues (Hosgood et al., 2014; Tsay et al., 2018). It is then a natural question whether there exists a dysbiosis of the gut microbiome in patients with LC. Studies of the gut microbiome composition in patients with LC and analysis of the effects of the gut microbiome on LC are urgently needed. Therefore, we tested whether LC patients differ in gut microecology when compared to healthy controls.

## Materials and Methods

### Study Participants

A total of 60 fecal samples were collected from 30 LC patients (median age: 61) and 30 matched healthy controls from the Department of Respiration, The Third Affiliated Hospital of Harbin Medical University (Table 1). All the LC patients were diagnosed according to their histopathological features using tumor node metastasis (TNM) scale classification of malignant tumors after surgery. The patients and healthy controls had not taken any medications in the 3 months before specimen collection. Informed consent was obtained from all participants. The fecal specimens were frozen in liquid nitrogen immediately after sampling and stored in a −80°C freezer.

**Table 1:** Characteristics of the study groups.

**Characteristics**	**Control (*n* = 30)**	**LC (*n* = 30)**	***P*-value**
**SEX**, ***n*** **(%)**			
Females	20 (66.7)	18 (60.0)	
Males	10 (33.3)	12 (40.0)	0.316
**AGE (YEARS)**			
Median	50	61	0.062
Range	19–95	52–72	
**PATHOLOGICAL CLASSIFICATION (%)**			
Small-cell lung cancer		7 (23.3)	
Non-small-cell lung cancer		23 (76.7)	
**CLASSIFICATION BY TNM STANDARD (%)**			
I B		1 (3.3)	
II A		3 (10.0)	
II B		1 (3.3)	
III A		17 (56.7)	
III B		8 (26.7)	

*There were no significant differences in the age or sex of the two groups according to the Mann-Whitney U-test; LC, lung cancer; TNM, tumor node metastasis scale*.

### DNA Extraction and PCR Amplification

Total DNA extraction from the samples was conducted according to the instructions of the OMG-soil kit (Omega Bio-tek, Norcross, GA, USA). DNA concentration and purity were determined using NanoDrop2000 (Thermo Fisher Scientific, Waltham, MA, USA), and the quality of the extracted DNA was inspected by 1% agarose gel electrophoresis. PCR amplification of the V3-V4 variable region was performed using 338F (5′-ACTCCTACGGGAGGCAGCAG-3′) and 806R (5′-GGACTACHVGGGTWTCTAAT-3′) primers by the following amplification procedure: pre-denaturation at 95°C for 3 min, 27 cycles (denaturation at 95°C for 30 s, annealing at 55°C for 30 s, extension at 72°C for 30 s), and extension at 72°C for 10 min (PCR: ABI GeneAmp^®^ 9700). The amplification was conducted in a 20 μL volume containing 4 μL of 5 × FastPfu buffer, 2 μL of 2.5 mM dNTPs, 0.8 μL of primer (5 μM), 0.4 μL of FastPfu polymerase, and 10 ng of DNA template.

### Illumina Miseq Sequencing

The PCR products were recovered using a 2% agarose gel, purified using an AxyPrep DNA Gel Extraction Kit (Axygen Biosciences, Union City, CA, USA), eluted with Tris-HCl, and detected by 2% agarose electrophoresis. Quantification was performed using QuantiFluorTM-ST (Promega, Madison, WI, USA). The purified amplified fragment was included in a library of PE 2 × 300 according to the standard operating protocol of the Illumina MiSeq platform (Illumina, San Diego, CA, USA).

The steps of library construction were as follows: (1) connecting the “Y” shaped joint, (2) removing the self-ligated fragments using magnetic beads, (3) enriching the library by PCR amplification, and (4) denaturing DNA by sodium hydroxide. Sequencing was carried out on the Illumina Miseq PE300 platform (Shanghai Meiji Biomedical Technology Co., Ltd, Shanghai, China), and the raw data were uploaded to the NCBI database.

### Data Processing

The raw sequence data were handled using Trimmomatic software and spliced using the FLASH software as follows:

1. We first set a 50-bp window. If the average quality value in the window was lower than 20, all sequences at the back end of the base were truncated from the front-end position of the window, and then the sequences having a length <50 bp after the quality control were removed.
2. According to the base overlap, the sequences of the two ends were spliced, and the maximum mismatch rate between overlaps was set to 0.2, with a length >10 bp. All remaining sequences were abandoned.
3. According to the barcode and primers at both ends of the sequence, a sequence was assigned to a sample. In this process, the barcode had to be precisely matched, and the primer was allowed two base mismatches.

The UPARSE software (version 7.1 http://drive5.com/uparse/) was used to perform the clustering of operational taxonomic units (OTUs) on the sequences with 97% or greater similarity of the 16S rDNA sequences and to remove single sequences and chimeras during the clustering. Each sequence was annotated with a species classification using the RDP classifier (http://rdp.cme.msu.edu/), and the alignment threshold was set to 70% compared to the Silva database (SSU123).

### Alpha/Beta Diversity Analysis and Taxonomic Plots

Alpha diversity analysis was used to investigate bacterial species diversity in gut ecosystems between the LC group and healthy controls. Information, such as species abundance, was obtained by observing various index values like Chao, Shannon, Ace, and Simpson, and then a statistical *t*-test was used to detect whether the index value between the two groups was significantly different. Here, we selected the Shannon index as a metric to analyze the community richness and evenness between the two groups. Inter-group comparisons were performed using a Wilcoxon rank sum test of non-parametric data. A *t*-test was applied after the results were reflected as visual metrics using a histogram.

Beta diversity analysis represents a comparison of microbial community composition and is used here to assess differences between microbial community composition. The basic output of this comparison is a distance matrix that represents the difference between every two samples in the community. NMDS analysis (non-metric multidimensional scaling) was chosen for the sample similarity comparison between the LC group and the healthy controls. This is a method of simplifying, analyzing, and categorizing research objects (samples or quantities) in a multidimensional space into low-dimensional spaces, while retaining a method for analyzing raw relational data between objects. The basic feature is to regard the similarity or dissimilarity data between objects as a monotonic function of point distance. On the basis of maintaining the original data order relationship, the original data are replaced with new identical data columns for metric multidimensional scaling analysis. This approach simplifies the study objects (samples or quantities) in a multidimensional space into low-dimensional spaces for localization, analysis, and categorization, while preserving the original relationships between objects. The basic feature of NMDS is to regard the similarity or dissimilarity data between objects as a monotonic function of point distance and replace the original data with new identical data columns for metric multidimensional scaling analysis on the basis of maintaining the original data order.

To determine potential bacterial biomarkers that differ in abundance and occurrence between the LC group and healthy controls, LEfSe analysis in multi-level species was used (Puri et al., 2018). LEfSe is a software package for discovering high-dimensional biomarkers with inputs that include genes, metabolites, and classification. We first used the non-parametric factorial Kruskal-Wallis (KW) sum-rank test to detect specific species relating significant abundance differences in two groups. We then estimated the effect of each component (species) by LEfSe linear discriminant analysis (LDA). In order to detect the species contributing to the abundance differences in different groups of microbial communities, we carried out a test of significance differences between groups. Based on the obtained community abundance data, rigorous statistical methods were used to detect species with different richness in different groups (samples) of microbial communities, and hypothesis testing was performed to assess the significance of these observed differences.

### 16S Function Prediction Analysis

“16S function prediction analysis” was implemented to obtain functional information of the gut microbiome between LC patients and healthy controls (Ravi et al., 2018). We normalized the OTU abundance table by using PICRUSt (the PICRUSt software stores the COG (Clusters of Orthologous Groups of proteins) letter KO (KEGG Ontology) information corresponding to the greengene id) (Douglas et al., 2018), i.e., to remove the effect of the number of copies of the 16S marker gene in the species group. The COG family information corresponding to the OTU was obtained by using the GreenGene ID corresponding to each OTU. The abundance of each COG was calculated. According to the COG database information, the description information of each COG and its work information was parsed from the eggNOG database to obtain a functional abundance spectrum (Huerta-Cepas et al., 2016).

The functional abundance spectrum reflected different levels of expression of related functional proteins or specific metabolic capacity of microbiome. By combining the distribution of the research objects in various functional categories, we had the opportunity to make a conclusion for the role of gut microbiota in the development of LC.

### Implementation of Statistical Analysis

All statistical calculations were performed in R 3.4.3. The correction of the *P*-value is responsible for the false discovery rate (FDR).

## Results

### Raw Data Management

After curation of the sequences, a total of 2,682,019 sequence fragments were obtained from the 60 samples, with an average length of 433 bp. To facilitate the storage and sharing of high-throughput sequencing data generated in this work, we uploaded the original sequence file of 1,162,191,785 bp to the NCBI large-capacity database SRA (Sequence Read Archive, http://www.ncbi.nlm.nih.gov/Traces/sra Accession: PRJNA507734).

### OTU Clustering and Evaluation

We obtained 740 OTUs by statistical analysis of the 16S rDNA sequences at a 97% similarity level. The community composition of each sample was statistically analyzed at the taxonomical ranks of phylum, class, order, family, genus, and species (i.e., OTU here). We constructed a rarefaction curve based on the number of sequences drawn. The Shannon-Wiener curve tended to be flat, indicating that the sequencing depth was sufficient to reflect the microbial diversity in the sample (Figure S1).

No significant difference was observed in alpha diversity between LC patients and healthy controls.

**Figure 1:**
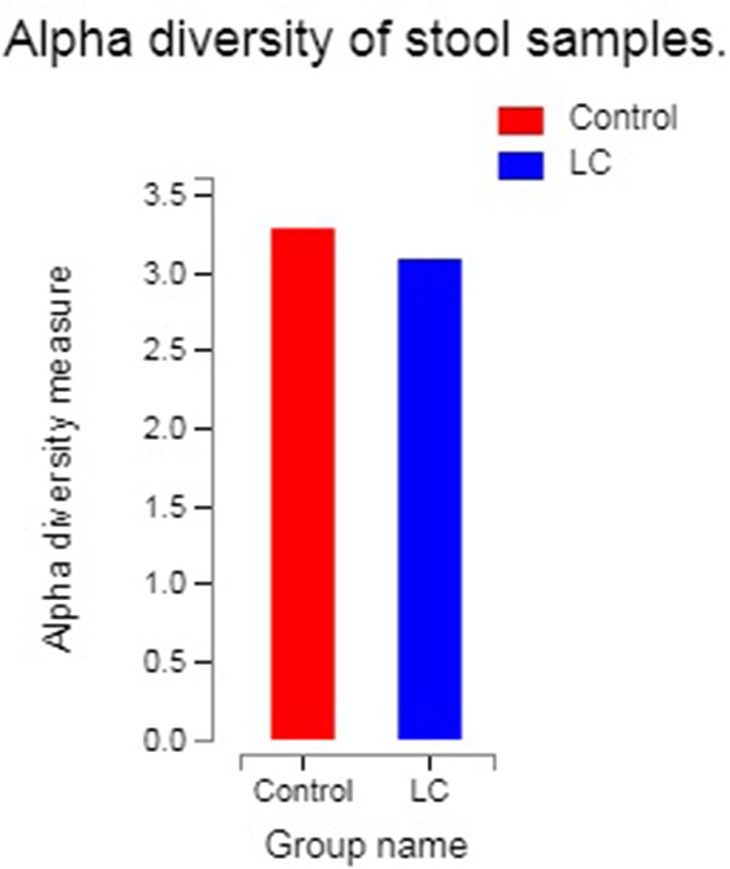
The abscissa is the group name, and the ordinate is the exponential average of each group (0.01 < *P* ≤ 0.05 marked as *, 0.001 < *P* ≤ 0.01 marked as **P ≤ 0.001 marked as***). LC: lung cancer.

The results of alpha diversity analysis were quantified by the Shannon index, which relates both the evenness and richness of a total of 740 OTUs obtained from the LC group and healthy controls. Figure 1 reflected the alpha diversity measurements for LC patients vs. healthy controls. The Shannon index of OTU level of healthy controls and LC patients were 3.28 and 3.09, respectively. Statistical testing using Welch's *t*-test showed no significant difference for Shannon diversity for the observed species (*P* observed = 0.1422).

### LC Patients and Controls Differ in Gut Microbial Composition

**Figure 2:**
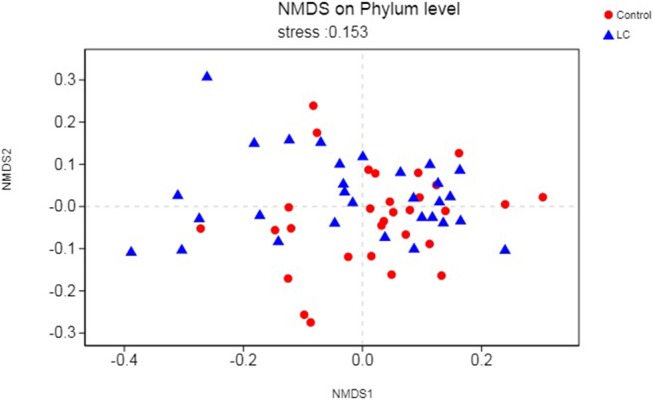
Points of blue colors or shapes represent LC samples; red colors or shapes represent samples of control. The closer the two sample points are, the more similar the composition of the two sample species is. The horizontal and vertical coordinates represent relative distances and have no practical significance. It is generally considered that stress <0.2 can be expressed by the two-dimensional dot pattern of NMDS, and its graph has a certain explanatory meaning. LC, lung cancer.

As a dimensionality reduction-based approach, NMDS (Non-metric multidimensional scaling) analysis was applied for dissimilarities in the microbial composition between LC patients and healthy controls (Noval Rivas et al., 2013). Results from NMDS are displayed in Figure 2. Patients with LC (blue dots) showed a shift to the left, which indicated compositional differences, and is measured by the NMDS intensity index (stress = 0.153 on phylum level). The separation intensity in other levels were as follows: class (stress = 0.16), order (stress = 0.146), family (0.153), and genus (0.21, unexplanatory meaning), the graphics of which are summarized in Figure S2.

### Specific Species in Multi-Level Tests

**Figure 3:**
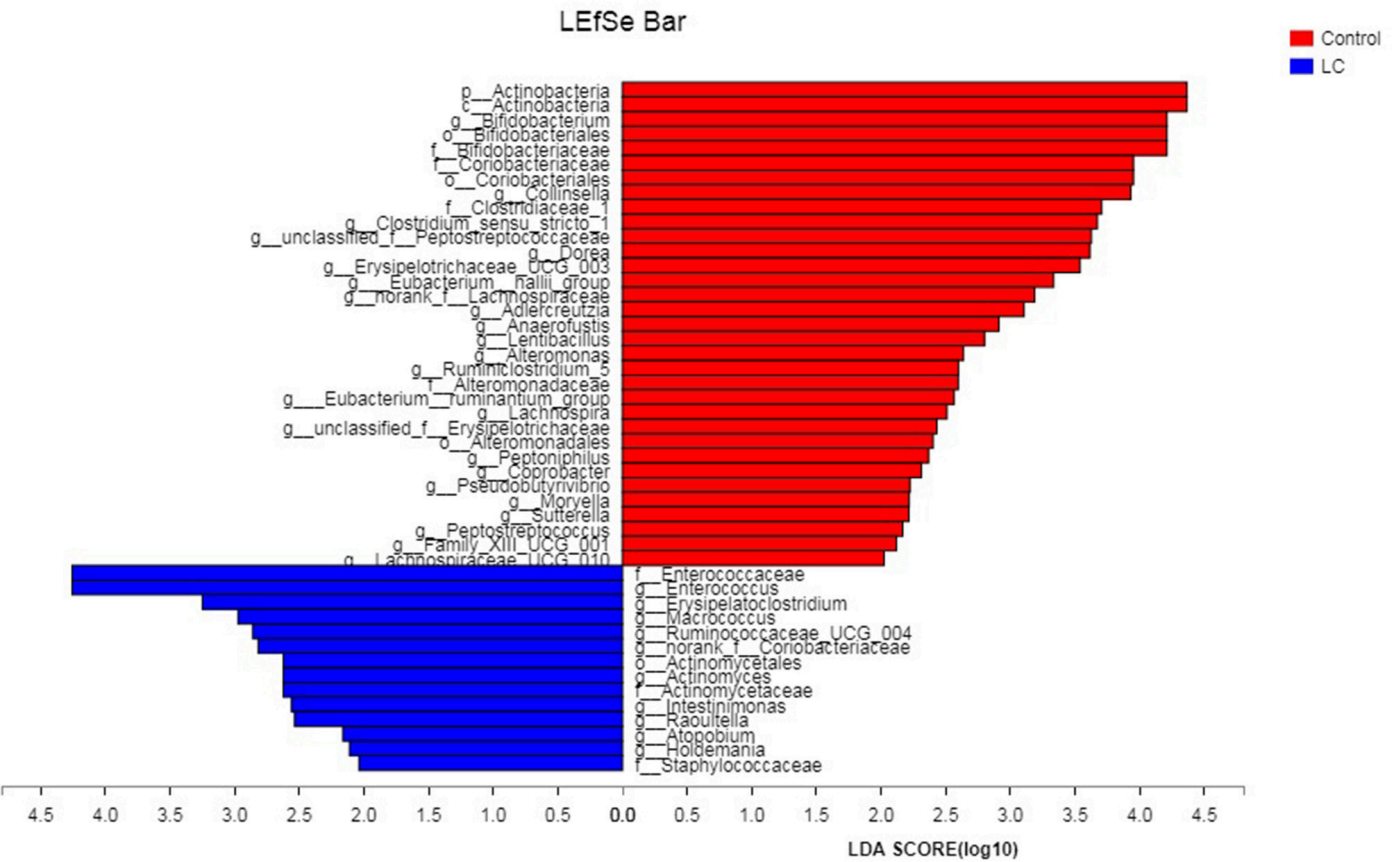
The LDA score obtained by linear regression analysis (LDA), the larger the LDA score, the greater the influence of species abundance on the difference effect. LC, lung cancer.

The multi-level LEfSe analysis for biomarkers was used to find significantly imbalanced species between LC patients and healthy controls, which showed substantially differentiated the two groups. LEfSe results illustrated 47 bacterial taxonomic clades having statistically significant differences (33 increased and 14 decreased) in LC patients. At the phylum level, increased *Actinobacteria* was detected as the strongest marker in healthy controls (Figure 3). Analysis at the class level also showed elevated levels of *Actinobacteria* in healthy individuals (Figure 3). At the order level, *Bifidobacteriales* showed a greater abundance in healthy controls (Figure 3). At the family level, increased bacteria such as *Bifidobacteriaceae* and *Coriobacteriaceae* were detected as markers in the control group and *Enterococcaceae* in the LC group (Figure 3). Numerous differential bacterial biomarkers were found at the genus level (Figure 3). Again, a multi-level Wilcoxon rank-sum test bar plot confirmed the above findings. A difference significance test between the two groups was based on the obtained community abundance data for hypothesis testing, and the significance of the difference was observed (Table 2, Figure S3). Combined with the results of the LEfSe test, the differences in species composition were judged mainly from *Actinobacteria* (*P* = 0.041)*, Bifidobacterium* (*P* = 0.012) and *Enterococcus* (*P* = 0.018; Table 2).

**Table 2:** Taxa differentially represented in the gut microbiomes of LC patients and healthy controls.

**Taxa**	**Control (%)**	**LC (%)**	***P*-value**
**Phylum**			
*Actinobacteria*	7.735	3.141	0.0406
**Class**			
*Actinobacteria*	7.735	3.141	0.0406
**Order**			
*Bifidobacteriales*	4.703	1.517	0.0138
*Coriobacteriales*	2.948	1.087	0.0351
**Family**			
*Bifidobacteriaceae*	4.703	1.517	0.0138
*Enterococcaceae*	0.226	4.258	0.0180
*Coriobacteriaceae*	2.948	1.087	0.0351
**Genus**			
*Bifidobacterium*	4.695	1.505	0.0121
*Enterococcus*	0.226	4.257	0.0187

*Significant difference between groups based on the obtained community abundance data by Kruskal-Wallis test; LC, lung cancer*.

### Differential Microbiome Functional Abundance Spectrum in the Lung Cancer Group

We implemented a 16S functional predictive analysis by COG (Cluster of Ortholog Genes) functional annotation, where the description information of each COG and its functional information was parsed from the eggNOG database. Compared to the healthy controls, results showed a significant decline in the functional abundance spectrum including 24 gut microbiota metabolic pathways in LC patients (Table 3). Among them, the expression of functional proteins involved in chromatin structure and dynamics and RNA processing and modification both decreased by more than 80%. At the same time, the only up-regulated was the extracellular structures related metabolic functions, which rose more than 10% in protein expression level (Table 3). The legends are shown in Figure S4.

**Table 3:** Functional abundance spectrum of gut microbiome in LC patients and healthy controls.

**Functional level classification**	**Control (median)**	**LC (median)**	**Difference ratio (%)**
Carbohydrate transport and metabolism	2959599	2691015	−9.07%
Function unknown	2731054	2507793	−8.17%
Amino acid transport and metabolism	2633394	2290167	−13.0%
Transcription	2610684	2422958	−7.19%
General function prediction only	2509370	2364836	−5.75%
Replicating, recombination and repair	2310377	2079278	−10.0%
Translation ribosomal structure and biogenesis	2115246	1879680	−11.1%
Cell wall/membrane/envelope biogenesis	2029903	1857081	−8.51%
Signal transduction mechanisms	1776253	1625486	−8.48%
Energy production	1856959	1624919	−12.4%
Inorganic ion transport and metabolism	1728393	1669573	−3.40%
Coenzyme transport and metabolism	1111055	982456	−11.5%
Defense mechanisms	1011090	940100	−7.02%
Nucleotide transport and metabolism	989752	902508	−8.81%
Posttranslational modification, protein turnover, chaperones	974889	884352	−9.28%
Lipid transport and metabolism	739863	701716	−5.15%
Cell cycle control, cell division, chromosome partitioning	480860	428783	−10.8%
Intracellular trafficking, secretion, and vesicular transport	460625	428402	−6.99%
Cell motility	286766	239656	−16.4%
Secondary metabolites biosynthesis, transport and catabolism	233283	224935	−3.57%
Cytoskeleton	3971	3581	−9.82%
Chromatin structure and dynamics	8731	1710	−80.4%
RNA processing and modification	8158	1061	−86.9%
Extracellular structures	766	852	11.2%

*Difference ratio (%) = (Control (median) - LC (median))/ Control (median); LC, lung cancer*.

## Discussion

In this study, we found that patients with LC had no difference in gut microbial alpha diversity but showed significant differences in microbial composition compared to healthy controls (Figures 1, 2; Table 2). At the phylum level, we found that these differences were mainly caused by *Actinobacteria* (Table 2). At the genus level, *Bifidobacterium* and *Enterococcus* were found to be the highest potential biomarkers for lung carcinogenesis (Table 2). We also observed a differential microbiome function abundance by 16S function prediction between these two groups (Table 3, Figure S4).

These results are consistent with the growing body of evidence for a bidirectional relationship between the gut microbiome and malignancies (Eun et al., 2014; Flemer et al., 2017; Allali et al., 2018). Meanwhile, human studies have concluded that the metabolic processes and products of the gut microbiome regulate human health and disease, including the development of immune function, incidence of obesity or anorexia nervosa, various types of cancer (Kluyver, 1932; Belcheva et al., 2015; Takiishi et al., 2017; Zhou and Fang, 2018). At the level of the phylum, we found drastically reduced *Actinobacteria* sp. as a possible LC-associated biomarker. This phylum contains a large proportion of commensal species, which are part of the healthy human microflora (Kundu et al., 2017). Although our method does not allow the identification of specific species within *Actinobacteria*, the abundance reduction in this phylum may be involved in the pathogenesis of LC. If there is a causal role in LC, then cancer suppression of malignant cells by secondary metabolites of the gut actinomycetes might be of interest (Rangan and Hang, 2017). A study has recently obtained initial evidence to support this hypothesis: an isolated *Actinobacteria* sp. from healthy children had potent cancer-suppressing activities due to its secondary metabolites (Zhou et al., 2017). Ravikumar et al. found anticancer properties of sediment actinomycetes against MCF-7 and MDA-MB-231 cell lines (Ravikumar et al., 2012). Thus, anticancer compounds produced by specific *Actinobacteria* sp. had a direct killing effect on malignant cells, which might explain the observed overrepresentation of this genus in healthy samples. The LEfSe analysis and difference significance test both showed that the genus *Bifidobacterium* was significantly more abundant in controls, while the LC patients showed elevated levels of *Enterococcus*. Members of *Bifidobacterium* have a variety of probiotic functions, such as improving inflammatory bowel disease, ulcerative colitis, Crohn's disease, and colonic pouchitis (van den Broek et al., 2008; Wang et al., 2014; Klemenak et al., 2015; Liang et al., 2018). At the same time, *Bifidobacterium* can inhibit the growth of spoilage bacteria and decompose carcinogens to achieve anti-cancer effects and reduce the inflammation caused by TNF-α and lipopolysaccharide (Boesten et al., 2011; Klemenak et al., 2015; Lim and Kim, 2017). Recently, researchers such as Shang et al. have confirmed that TNF-α promotes metastasis of lung cancer by inducing epithelial–mesenchymal transition (Shang et al., 2017). If there is a causal relationship, the decrease in genus *Bifidobacterium* we find in LC patients may reflect the bacterial community involved in a progression of lung cancer. However, compared with *Bifidobacterium, Enterococcus* can produce many harmful chemicals that lead to increased DNA mismatch rate, which, in turn, causes genetic activity that promotes rectal cancer (Strickertsson et al., 2013; Amarnani and Rapose, 2017). In previous studies, *Enterococcus faecalis* infections have been confirmed to lead to NF-κB inflammatory responses and DNA damage. At the same time, the superoxide secreted by *Enterococcus* spp. sent a strong signal to macrophages, changed the growth and division of intestinal parasitic cells, and enhanced the activity of oncogenes (Strickertsson et al., 2013; Wang et al., 2015; Amarnani and Rapose, 2017). Although the underlying mechanisms of an inflammatory response-associated severity of lung carcinogenesis are not understood, the overrepresentation of *Enterococcus* has been suspected as being a missing link.

Using the 16s functional predictive analysis, significant differences in microbiome functional abundance between the two groups were observed. The healthy controls had a significantly higher microbiome functional spectrum, while the LC patients showed elevated levels only in expression of extracellular structures functional proteins. The various metabolic abilities of the gut microbiome are being reduced during the disease (Table 3). In the gut micro-ecology of LC patients, expressions of functional proteins involved in amino acid transport and metabolism, coenzyme transport and metabolism, cell cycle control, and cell division all decreased by more than 10%, which directly negatively impacted the reproduction of, and colonization by, gut bacteria. The gut microbiome is not only an important participant in the digestive function of the human body, but also plays a substantial role in maintaining the gut—the largest immune organ (Kau et al., 2011; Hwang et al., 2012; Zitvogel et al., 2017). A team led by Dr. Laurence Zitvogel of the Gustave Roussy Cancer Center showed that some cancer treatments rely on the gut microbiota to activate the immune system (Gopalakrishnan et al., 2018; Zitvogel et al., 2018). A large body of evidence confirms that the host and the enteric microbiome complement each other, and the disorder of the gut microecology is exacerbated during the disease state, which greatly impairs its beneficial functions (Kelly et al., 2015; Ringel, 2017). Undoubtedly, changes in microbial-associated molecular patterns and microbiome functions caused by bacterial dysbiosis are key pathways for the progression of LC.

The importance of the human gut microbiome is being increasingly recognized. As an important “organ” that has long been neglected, how to maintain a healthy gut microbiome is increasingly becoming a focus. However, due to the limitations in technical conditions and a lack of sufficient clinical data, the specific mechanisms of microbial-associated molecular patterns and bacterial metabolites driving cancer have not been fully elucidated. In the future, we will review in detail therapeutic modalities and discuss clinical settings in which targeting the “gut-microbiota–lung axis” for the prevention of LC seems promising. In addition, since the main research object of gut microbial diversity analysis is gut bacteria, the whole process of the experiment was carried out using bacterial universal primers for the amplification of bacterial marker genes, the virus and mycoplasma present in a small part were not analyzed. It is necessary to design a completion plan for this limitation in the future study.

In conclusion, compared to previous analyses of the relationship between LC and the lung microbiome, this is the first study of LC in connection with the gut microbiome (Hosgood et al., 2014; Kosikowska et al., 2016). Our findings support the hypothesis of LC-specific microbiota. We suggest that the reduced levels of *Actinobacteria* sp. and *Bifidobacterium* sp. and elevated levels of *Enterococcus* sp. are associated with LC. At the same time, we have further revealed that the damage of the normal function of the gut microbiome is associated with the progression of LC. the progress of LC. We hope that the results herein can provide some guidance for using gut microbes as biomarkers to assess the progression of lung cancer, or lead to interventional targets to control the development of the disease.

## Ethics Statement

Fecal samples were collected in this research without any medical expense or suffering by the subjects, and the results were used for scientific research purposes. We declare that none of the authors have any conflicts of interest or financial ties to disclose. This study was carried out in accordance with the recommendations of Ethical Guidelines for Biomedical Research, Harbin Medical University Ethics Review Committee (an affiliated department of Harbin Medical University). The protocol was approved by the Harbin Medical University Ethics Review Committee. All subjects gave written informed consent in accordance with the Declaration of Helsinki. The subjects' rights were adequately protected, and there was no potential risk to the subjects.

## Author Contributions

HZ, YW, LC, and Y-KZ are responsible for sequencing, article writing. M-FZ, G-DL, M-CZ, Y-GL, J-BZ, and Y-NG are responsible for the collection and processing of clinical samples. Y-JZ and S-LL are responsible for the experimental design.

### Conflict of Interest Statement

The authors declare that the research was conducted in the absence of any commercial or financial relationships that could be construed as a potential conflict of interest.

## Supplementary Material

Figure S1 The abscissa is the amount of sequencing data randomly selected; the ordinate is the number of species observed.

Figure S2 Points of blue colors or shapes represent LC samples; red colors or shapes represent samples of control. The closer the two sample points are, the more similar the composition of the two sample species is. The horizontal and vertical coordinates represent relative distances and have no practical significance. It is generally considered that stress <0.2 can be expressed by the two-dimensional dot pattern of NMDS, and its graph has a certain explanatory meaning. LC, lung cancer.

Figure S3 The axis represents the taxa name at a given classification level, and the column length corresponding to the species indicates the average relative abundance of the species in each sample group. The different colors indicate different groupings. The rightmost side is the *P*-value from Kruskal–Wallis test, ^*^ 0.01 < *P* ≤ 0.05, ^**^ 0.001 < *P* ≤ 0.01, ^***^ *P* ≤ 0.001. LC, lung cancer.

Figure S4 The abscissa is the first-level classification of COG function. LC, lung cancer.
