# Supplementary figures and images for: Dysbiosis of the Gut Microbiome in Lung Cancer
Source: Front Cell Infect Microbiol. 2019 Apr 18;9:112. doi: 10.3389/fcimb.2019.00112 (PMC6489541; doi:10.3389/fcimb.2019.00112)

Rarefaction curves

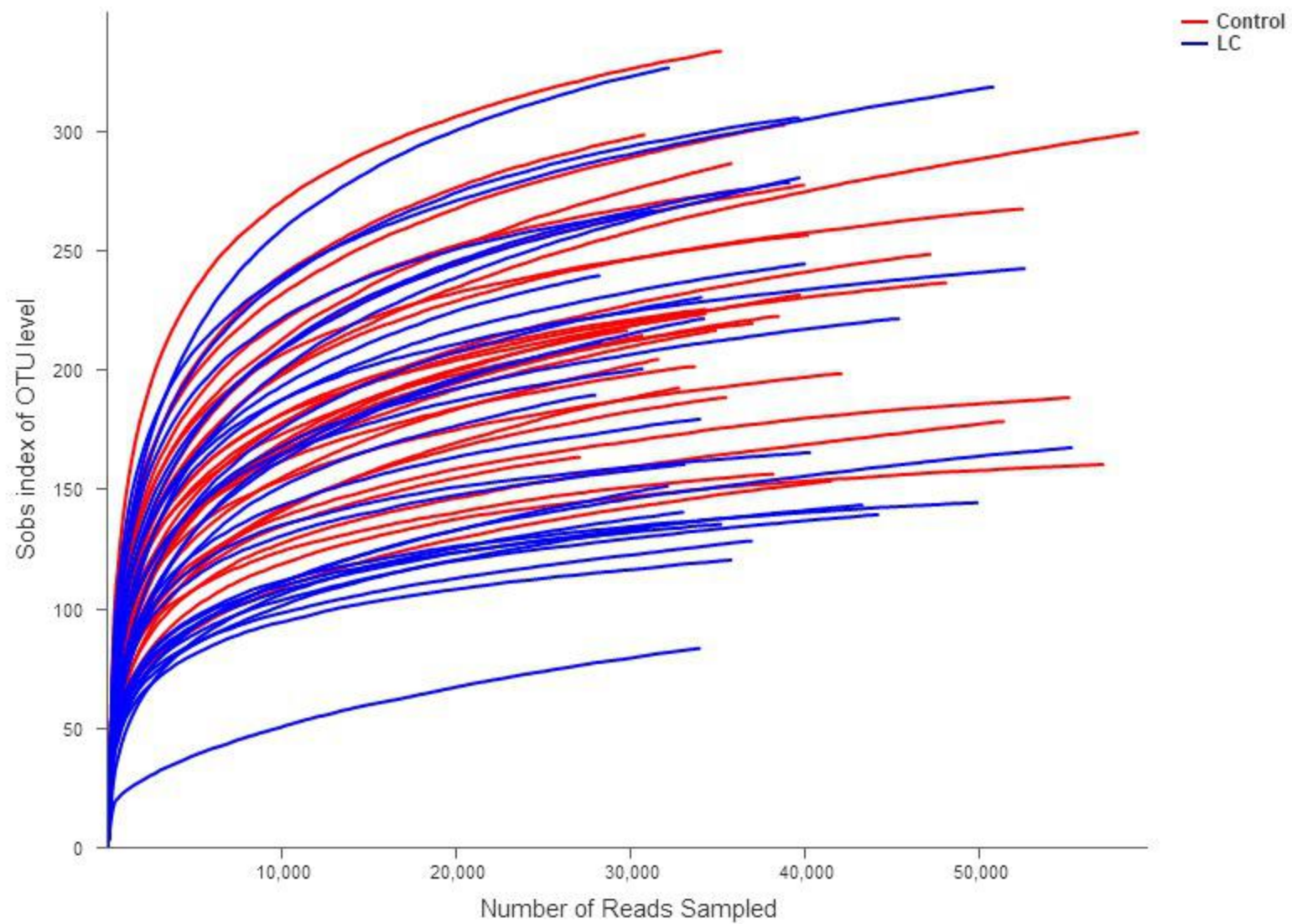

Supplement: Figure S1 — The abscissa is the amount of sequencing data randomly selected; the ordinate is the number of species observed. [file Data_Sheet_1.PDF]

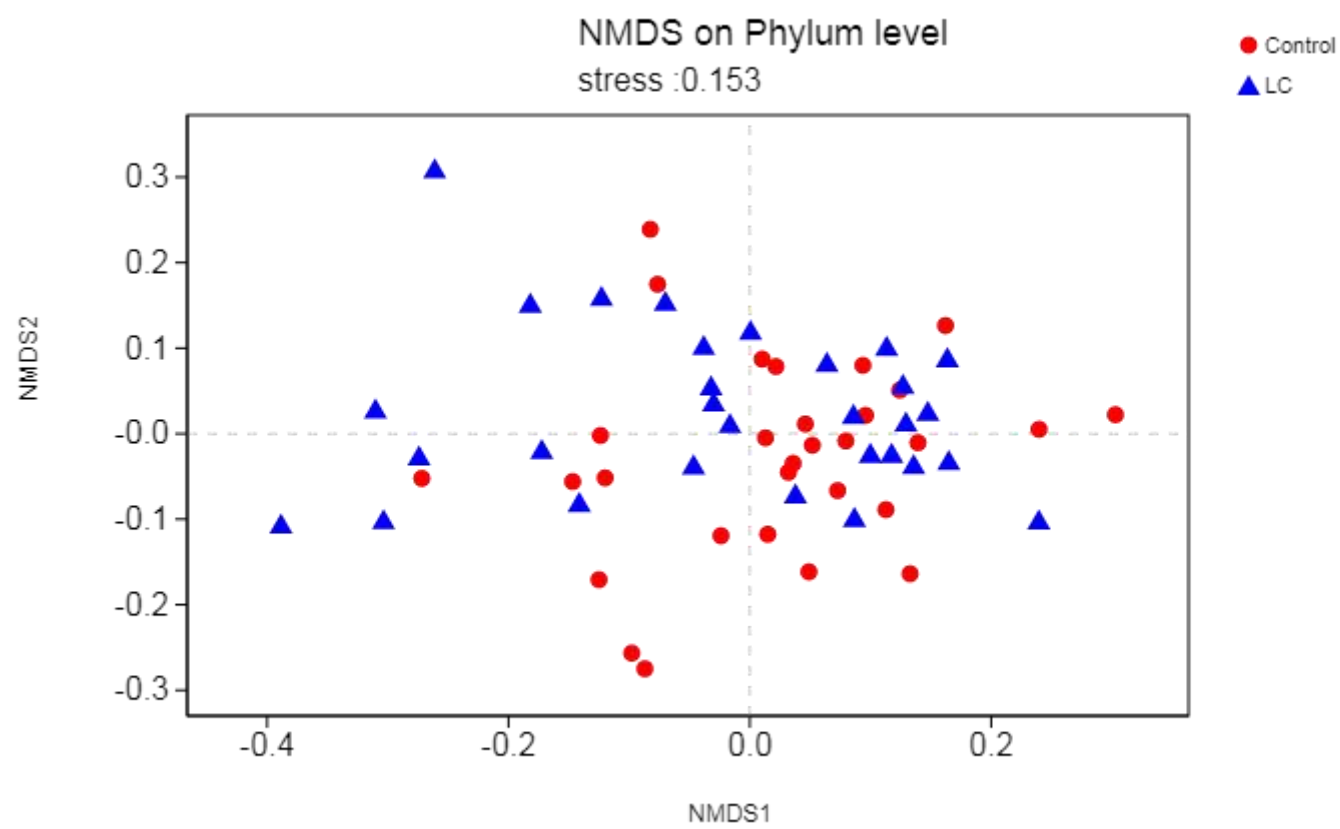

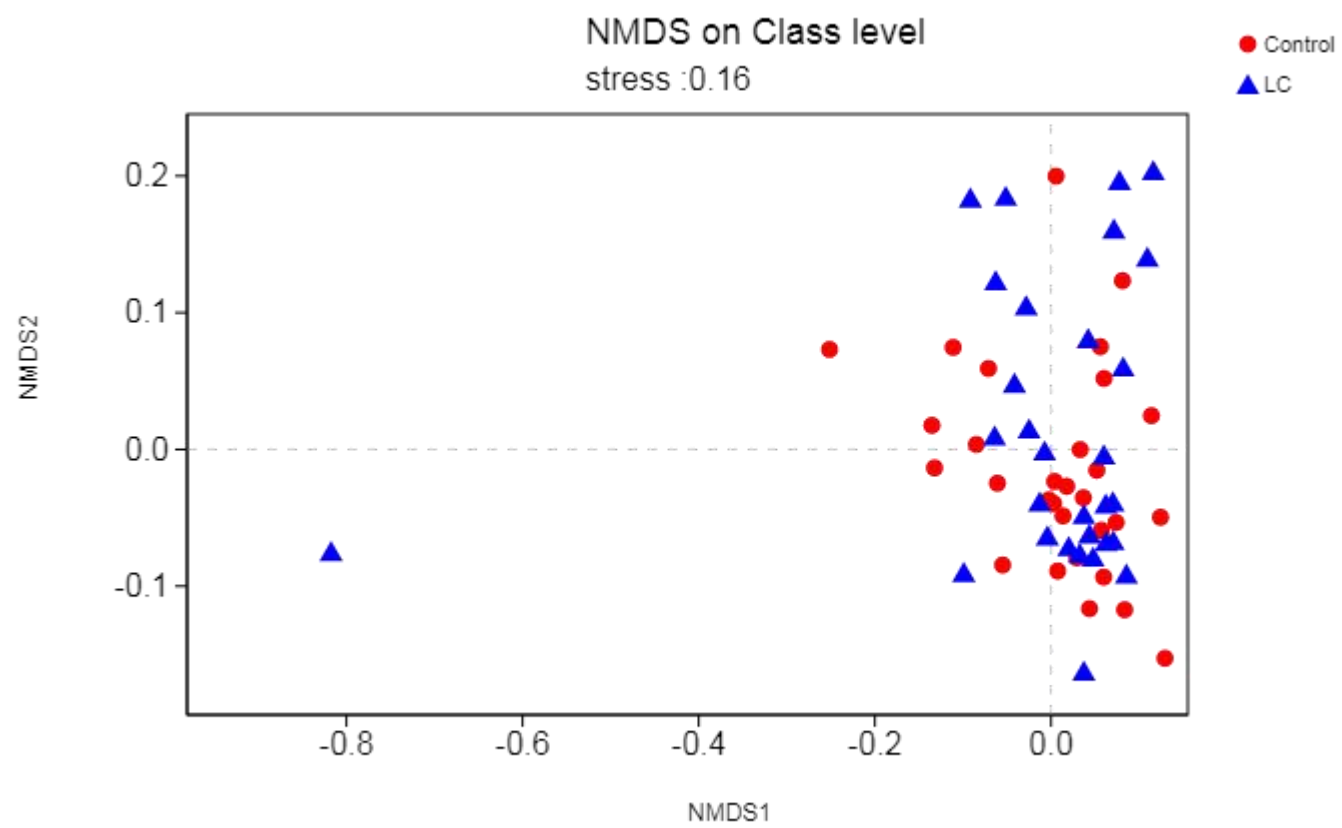

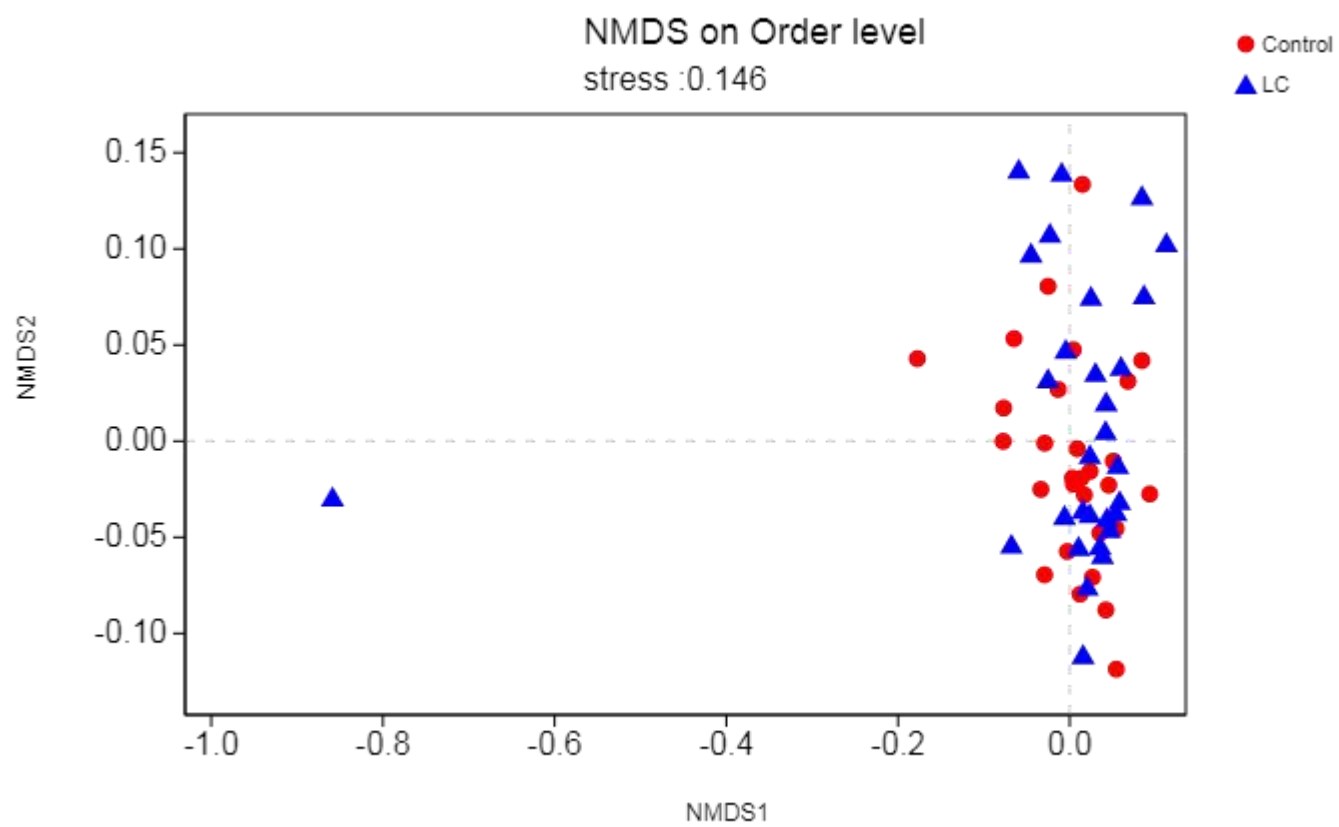

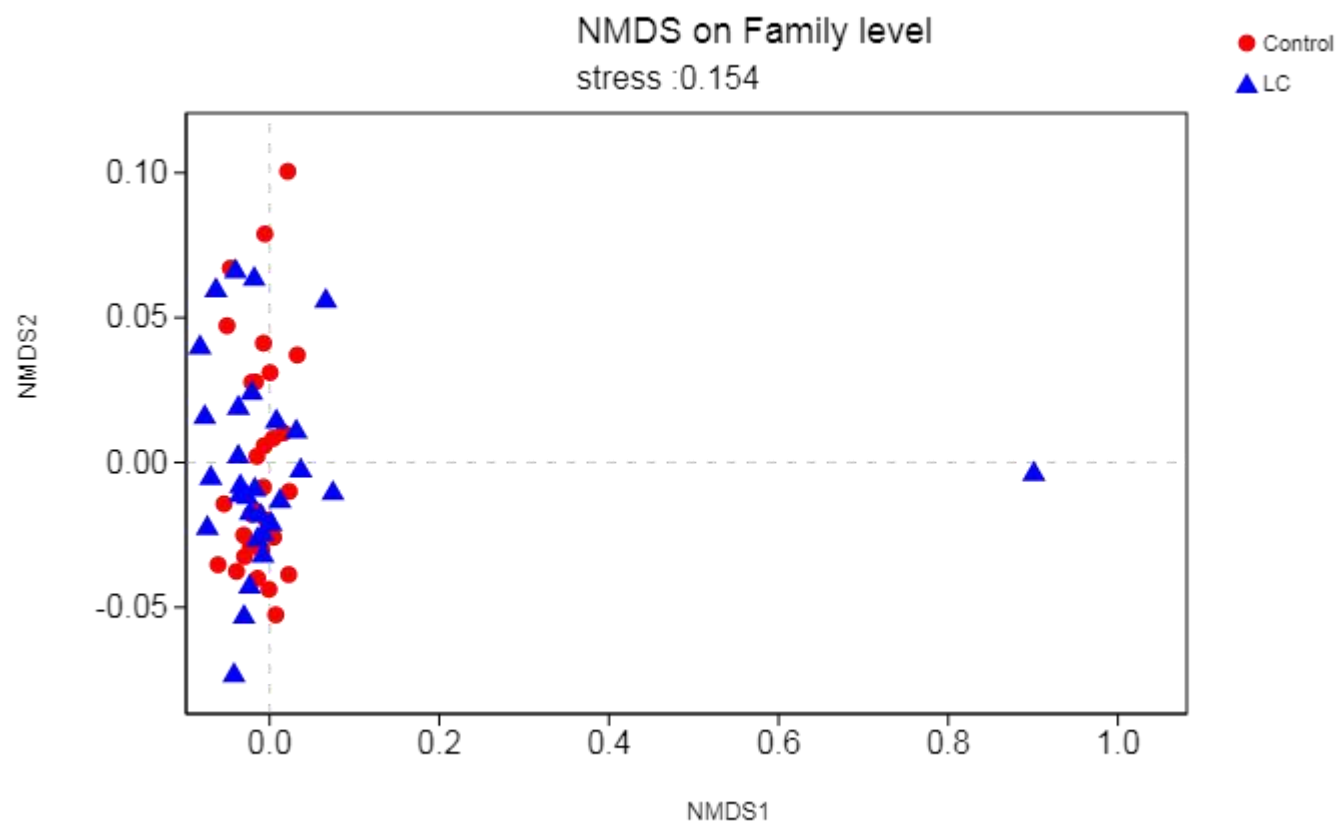

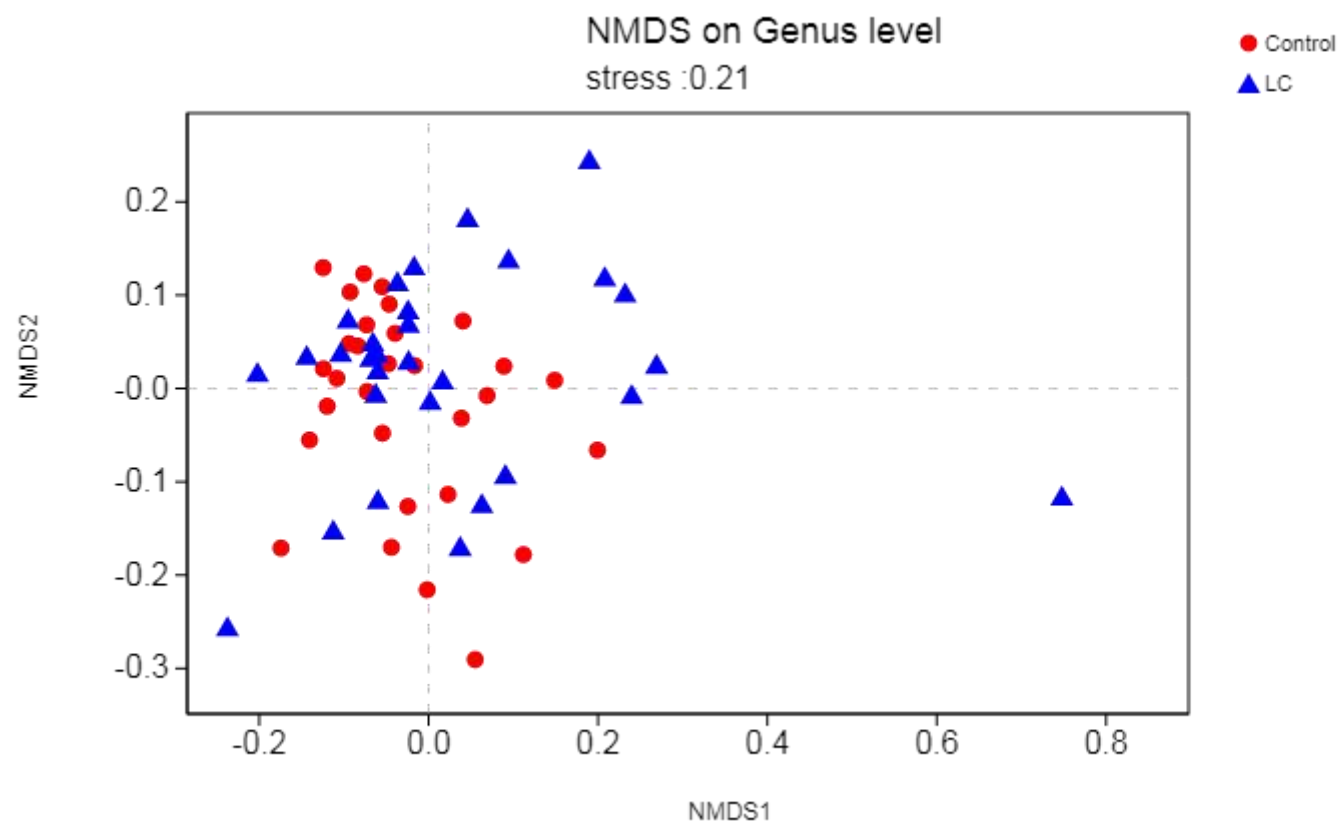

Supplement: Figure S2 — Points of blue colors or shapes represent LC samples; red colors or shapes represent samples of control. The closer the two sample points are, the more similar the composition of the two sample species is. The horizontal and vertical coordinates represent relative distances and have no practical significance. It is generally considered that stress <0.2 can be expressed by the two-dimensional dot pattern of NMDS, and its graph has a certain explanatory meaning. LC, lung cancer. [file Data_Sheet_2.PDF]

# COG function classification of the control

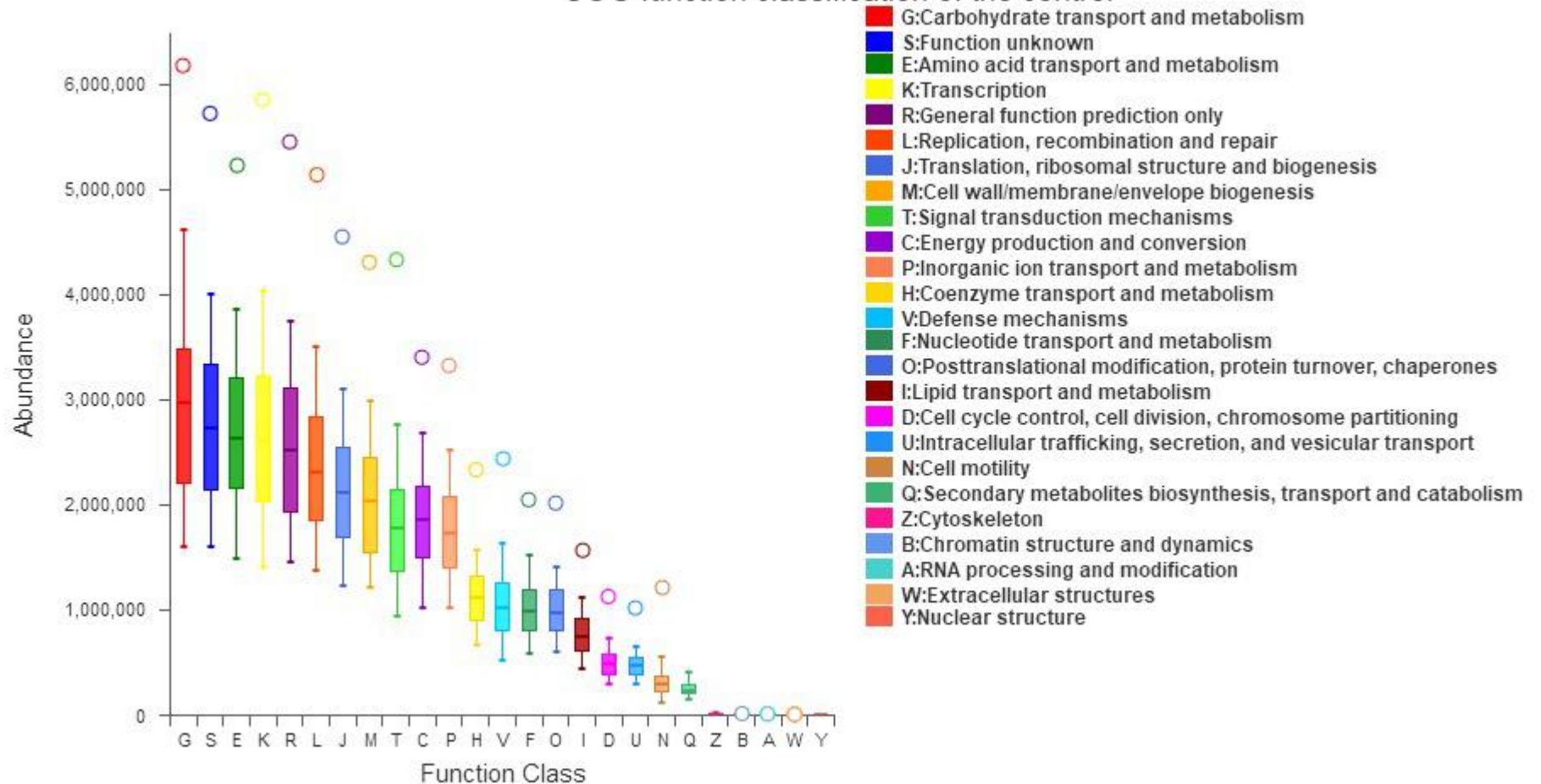

# COG function classification of the LC

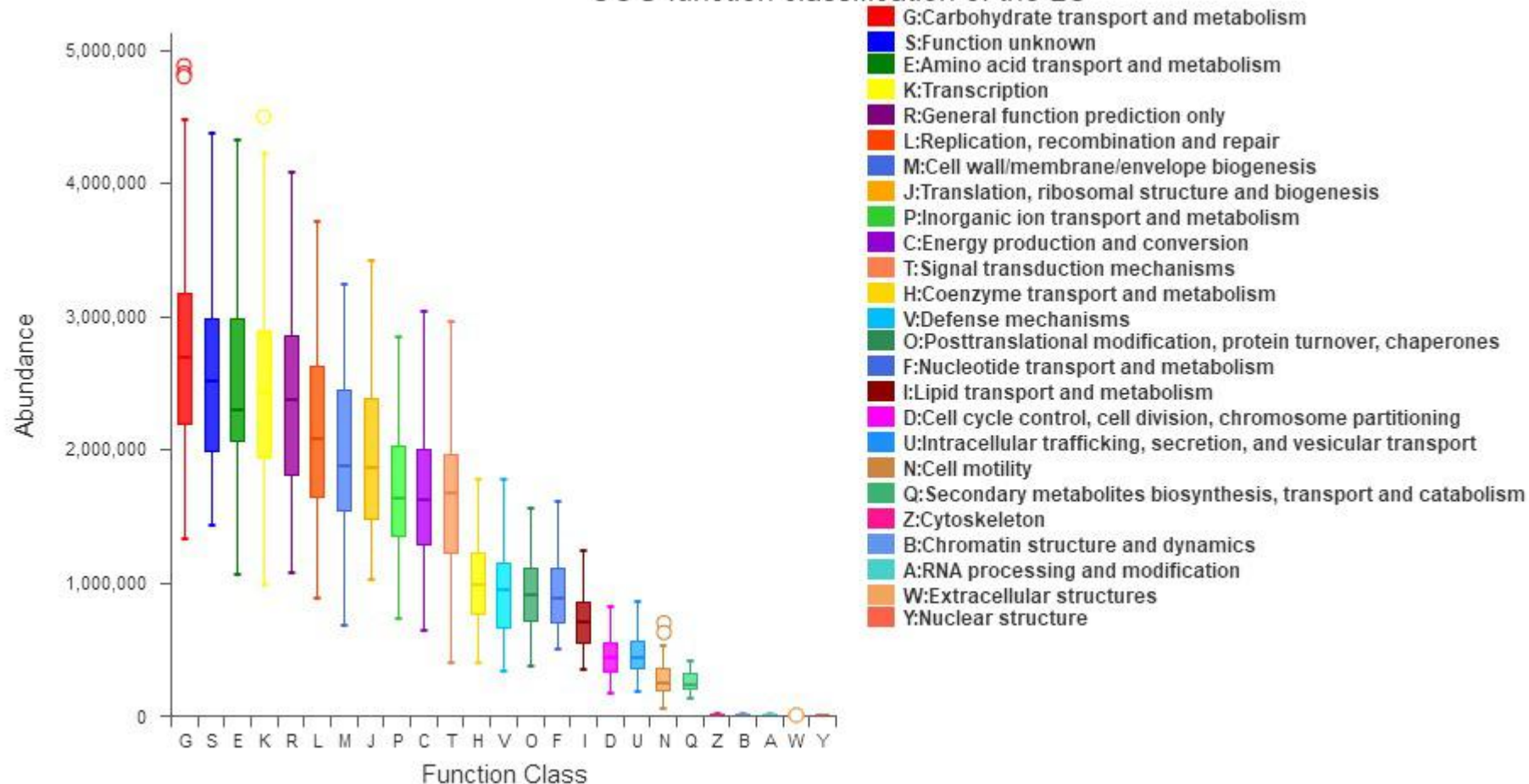

Supplement: Figure S4 — The abscissa is the first-level classification of COG function. LC, lung cancer. [file Data_Sheet_4.PDF]
