# Supplementary material for: Dysbiosis of the Gut Microbiome in Lung Cancer
Source: Front Cell Infect Microbiol. 2019 Apr 18;9:112. doi: 10.3389/fcimb.2019.00112 (PMC6489541; doi:10.3389/fcimb.2019.00112)
Supplement: Figure S3 — The axis represents the taxa name at a given classification level, and the column length corresponding to the species indicates the average relative abundance of the species in each sample group. The different colors indicate different groupings. The rightmost side is the P-value from Kruskal–Wallis test, * 0.01 < P ≤ 0.05, ** 0.001 < P ≤ 0.01, *** P ≤ 0.001. LC, lung cancer. [file Data_Sheet_3.PDF]

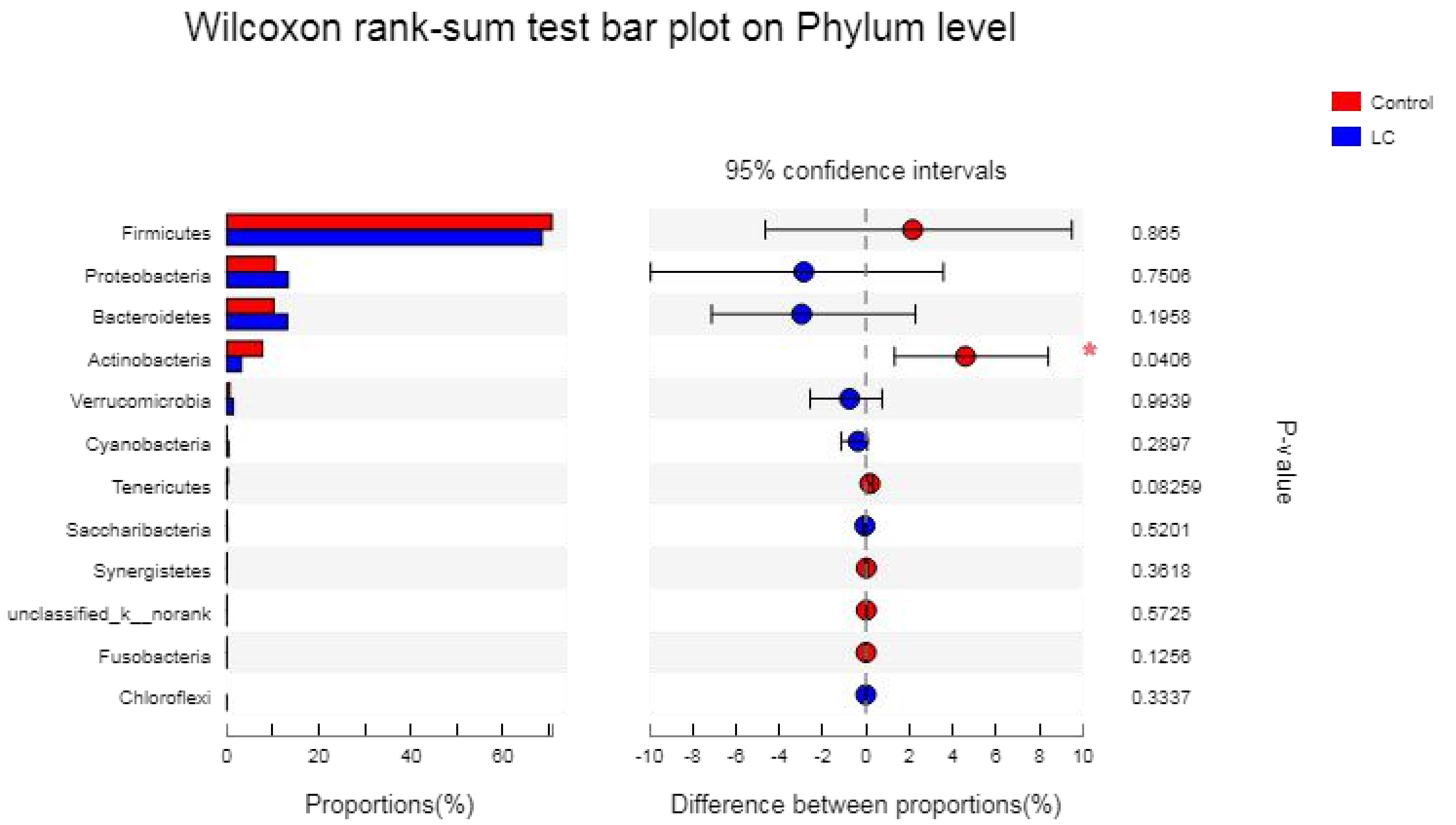

Wilcoxon rank-sum test bar plot on Phylum level
Control
LC
95% confidence intervals
Firmicutes
Proteobacteria
Bacteroidetes
Actinobacteria
Verrucomicrobia
Cyanobacteria
Tenericutes
Saccharibacteria
Synergistetes
unclassified_k__norank
Fusobacteria
Chloroflexi
0.865
0.7506
0.1958
*
0.0406
0.9939
0.2897
0.08259
0.5201
0.3618
0.5725
0.1256
0.3337
P-value
0
20
40
60
Proportions(%)
-10
-8
-6
-4
-2
0
2
4
6
8
10
Difference between proportions(%)

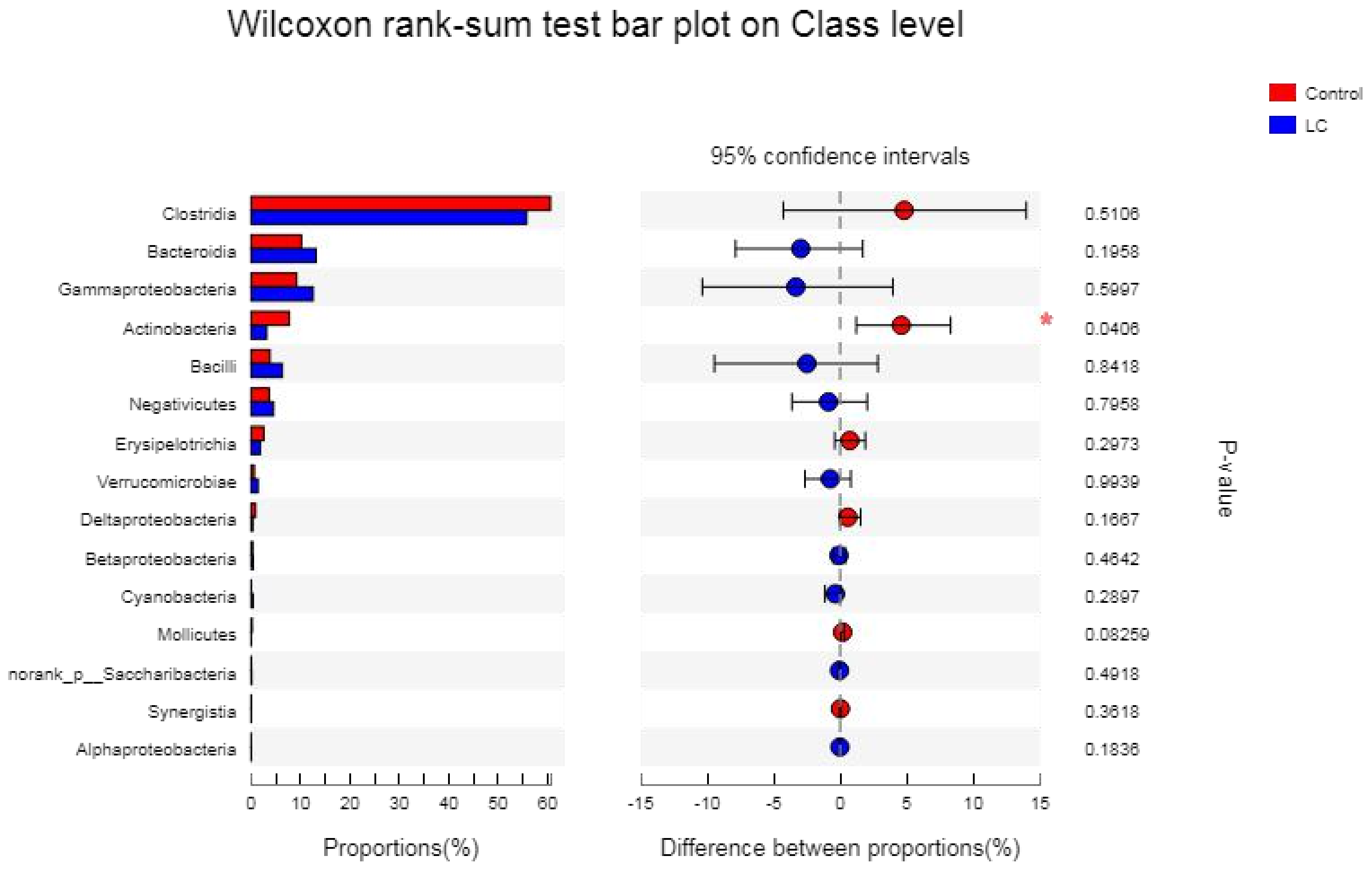

Wilcoxon rank-sum test bar plot on Class level
Control
LC
95% confidence intervals
Clostridia
Bacteroidia
Gammaproteobacteria
Actinobacteria
Bacilli
Negativicutes
Erysipelotrichia
Verrucomicrobiae
Deltaproteobacteria
Betaproteobacteria
Cyanobacteria
Mollicutes
norank_p__Saccharibacteria
Synergistia
Alphaproteobacteria
0.5106
0.1958
0.5997
*
0.0406
0.8418
0.7958
0.2973
0.9939
0.1667
0.4642
0.2897
0.08259
0.4918
0.3618
0.1836
P-value
0 10 20 30 40 50 60
Proportions(%)
-15 -10 -5 0 5 10 15
Difference between proportions(%)

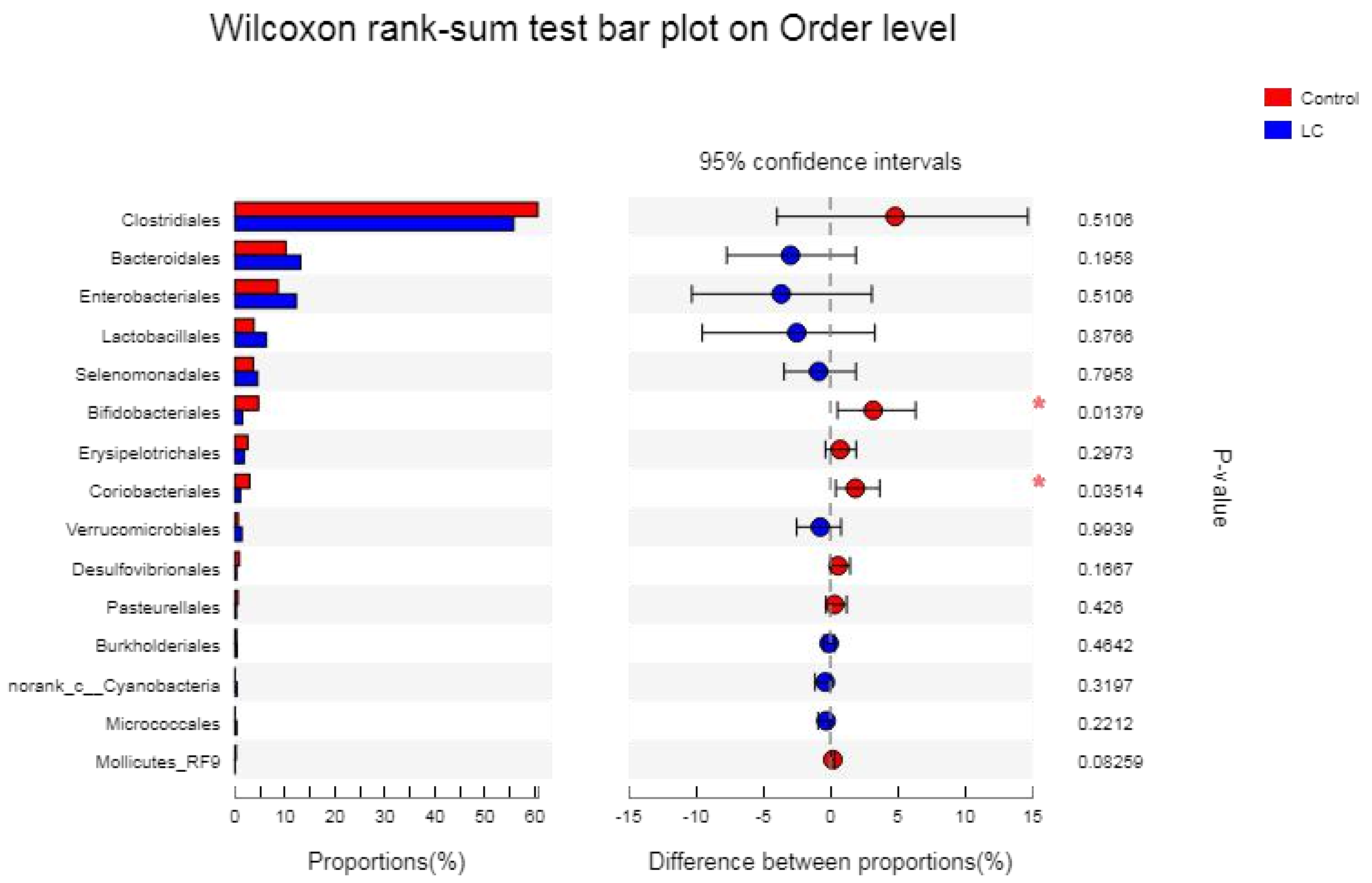

Wilcoxon rank-sum test bar plot on Order level
Control
LC
95% confidence intervals
Clostridiales
Bacteroidales
Enterobacteriales
Lactobacillales
Selenomonadales
Bifidobacteriales
Erysipelotrichales
Coriobacteriales
Verrucomicrobiales
Desulfovibrionales
Pasteurellales
Burkholderiales
norank_c__Cyanobacteria
Micrococcales
Mollicutes_RF9
0.5106
0.1958
0.5106
0.8766
0.7958
* 0.01379
0.2973
* 0.03514
0.9939
0.1667
0.426
0.4642
0.3197
0.2212
0.08259
P-value
0 10 20 30 40 50 60
Proportions(%)
-15 -10 -5 0 5 10 15
Difference between proportions(%)

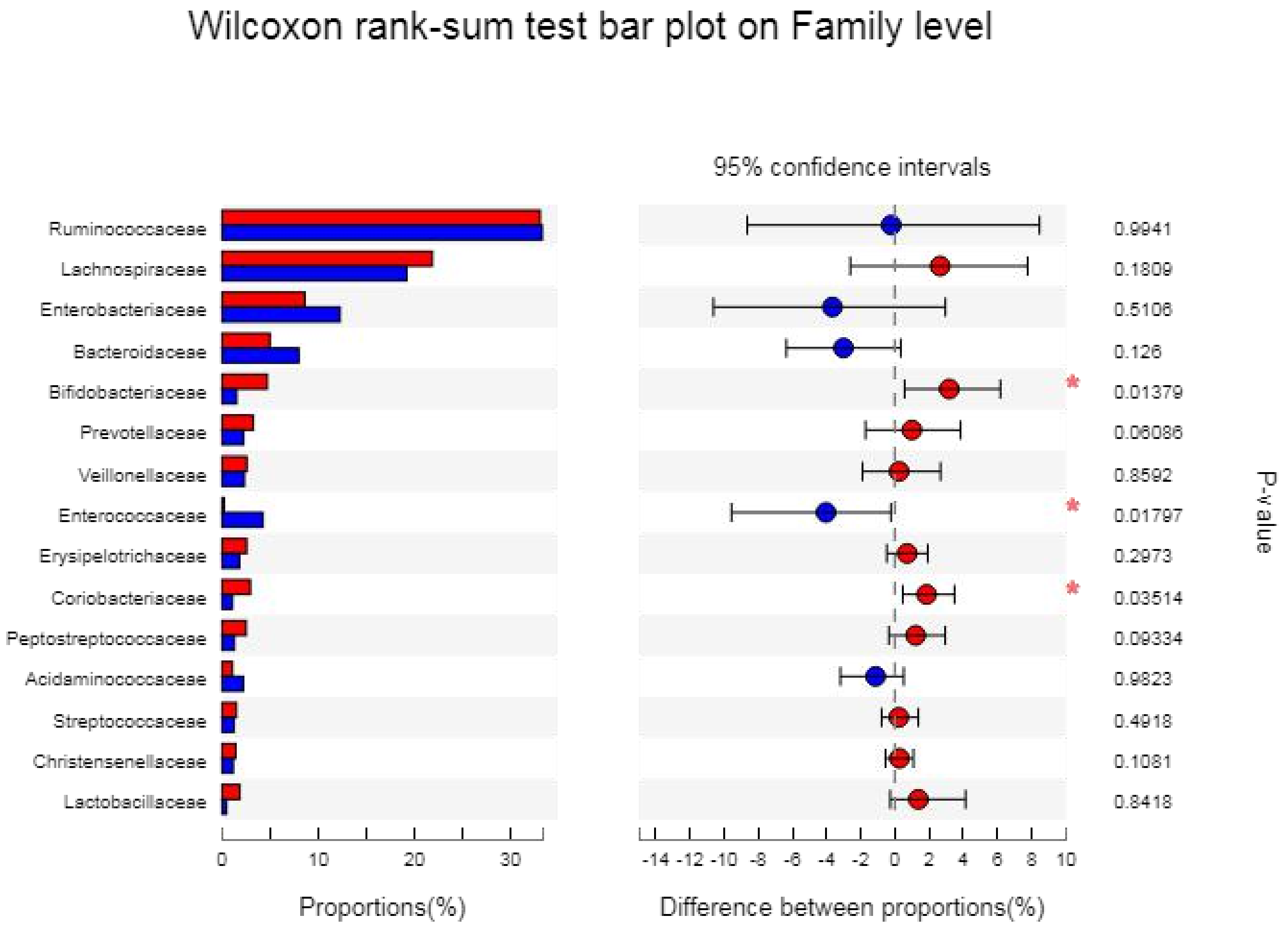

Wilcoxon rank-sum test bar plot on Family level
Control
LC
95% confidence intervals
Ruminococcaceae
Lachnospiraceae
Enterobacteriaceae
Bacteroidaceae
Bifidobacteriaceae
Prevotellaceae
Veillonellaceae
Enterococcaceae
Erysipelotrichaceae
Coriobacteriaceae
Peptostreptococcaceae
Acidaminococcaceae
Streptococcaceae
Christensenellaceae
Lactobacillaceae
0
10
20
30
Proportions(%)
-14 -12 -10 -8 -6 -4 -2 0 2 4 6 8 10
Difference between proportions(%)
0.9941
0.1809
0.5106
0.126
*
0.01379
0.06086
0.8592
*
0.01797
0.2973
*
0.03514
0.09334
0.9823
0.4918
0.1081
0.8418
P-value

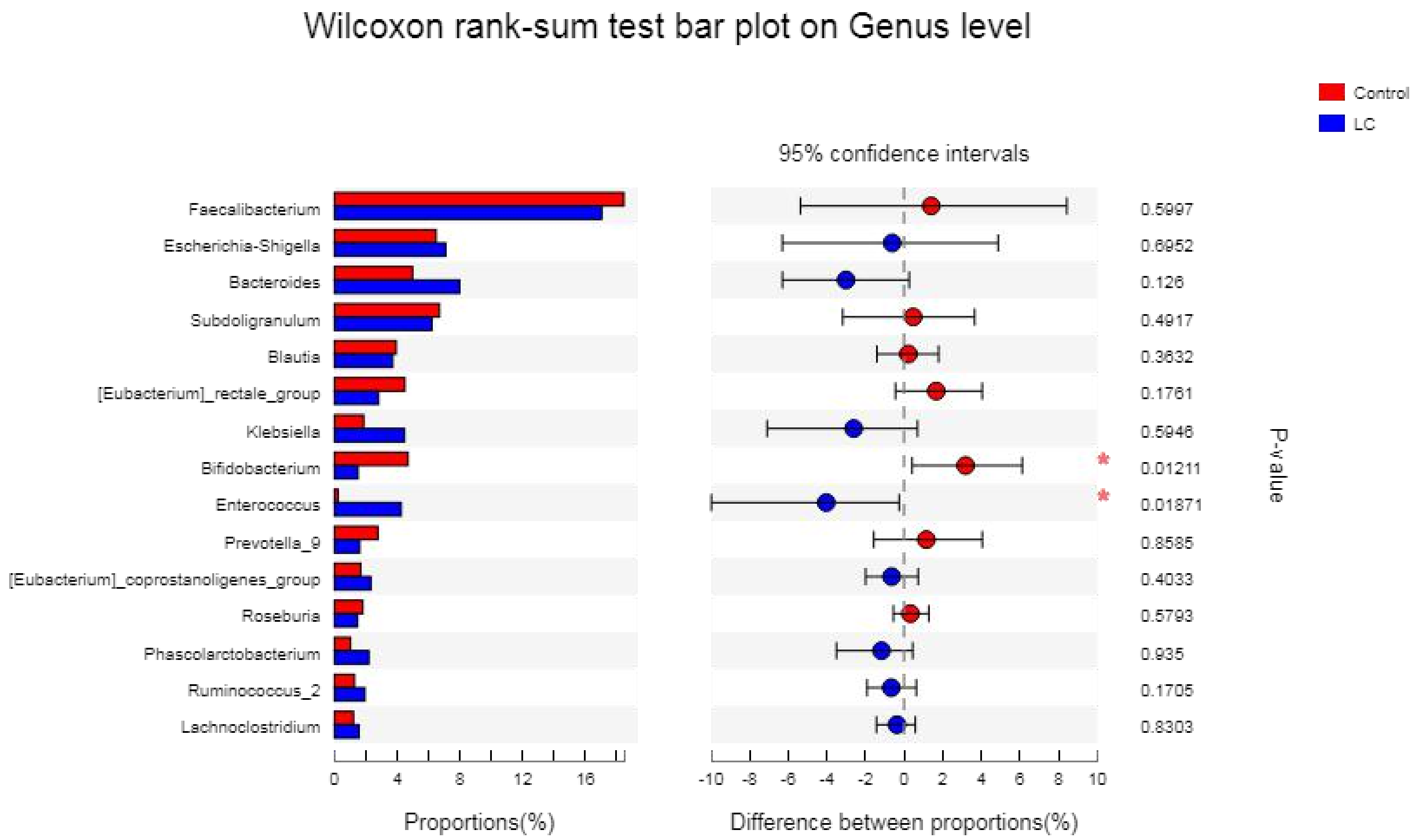

Wilcoxon rank-sum test bar plot on Genus level
Control
LC
95% confidence intervals
Faecalibacterium
Escherichia-Shigella
Bacteroides
Subdoligranulum
Blautia
[Eubacterium]_rectale_group
Klebsiella
Bifidobacterium
Enterococcus
Prevotella_9
[Eubacterium]_coprostanoligenes_group
Roseburia
Phascolarctobacterium
Ruminococcus_2
Lachnoclostridium
0.5997
0.6952
0.126
0.4917
0.3632
0.1761
0.5946
* 0.01211
* 0.01871
0.8585
0.4033
0.5793
0.935
0.1705
0.8303
P-value
0 4 8 12 16
Proportions(%)
-10 -8 -6 -4 -2 0 2 4 6 8 10
Difference between proportions(%)
